# Fostering university students’ autonomous motivation through a societal impact project: a qualitative study of students’ and teachers’ perspectives

**DOI:** 10.1186/s12909-024-06494-9

**Published:** 2024-12-20

**Authors:** Yuanyuan Zhu, Latifa Abidi, Hans Savelberg, S. Eleonore Köhler, Rashmi A. Kusurkar, Diana Dolmans

**Affiliations:** 1 Department of Educational Development and Research, School of Health Professions Education, Faculty of Health, Medicine and Life Sciences, Maastricht University, Maastricht, PObox 616, 6200MD The Netherlands; 2 Department of Health Promotion, Faculty of Health, Medicine and Life Sciences, Maastricht University, Peter Debyeplein 1, Maastricht, 6229 HA The Netherlands; 3 Research in Education, LEARN! Research Institute for Learning and Education, Faculty of Psychology and Education, Amsterdam Public Health, Quality of Care, Amsterdam UMC location Vrije Universiteit Amsterdam, De Boelelaan 1118, Amsterdam, 1081 HZ The Netherlands

**Keywords:** Autonomous motivation, Basic psychological needs, Self-Determination theory, Health professions education, Qualitative study, Curriculum design

## Abstract

**Background:**

Fostering students’ autonomous motivation is linked to numerous positive outcomes. However, stimulating autonomous motivation of students in health professions remains a challenge. According to the Self-Determination Theory, supporting students’ basic psychological needs for autonomy, relatedness, and competence fosters their autonomous motivation. However, there is a lack of studies that explore how and why education might enhance students’ autonomous motivation. We designed, implemented, and investigated an extracurricular project called the ‘Societal Impact Project’ (SIP) to support students’ basic psychological needs and autonomous motivation through three principles, offering authentic and collaborative learning experiences as well as scaffolding. This study aimed to understand how and why the SIP with characteristics of authentic and collaborative learning, and scaffolding supports students’ autonomy, relatedness, competence, and autonomous motivation from the students’ and teachers’ perspectives.

**Methods:**

First-year students following the bachelor programmes of Biomedical Sciences and Health Sciences participated in the project. Students and teachers took part in focus groups conducted after the project. We adopted thematic analysis.

**Results:**

Students reported that, firstly, having freedom was motivating, but students needed different adaptive degrees of guidance throughout the project. Secondly, working in small groups could be motivating or demotivating, but having peer connections and openly discussing difficulties made the groups strong. Thirdly, societal relevant problems stimulated motivation and learning as students recognized the real-life value of the problems, but the relevance of these problems to students’ curriculum was not always clear to them.

**Conclusions:**

SIP reflected characteristics of the three educational principles, and students reported that these elements contributed to student’s basic psychological needs and autonomous motivation. A careful balance is needed in terms of offering autonomy versus support. Furthermore, students faced difficulties in seeing the link between the societal relevant problems and their curricula.

**Supplementary Information:**

The online version contains supplementary material available at 10.1186/s12909-024-06494-9.

## Background

Autonomous motivation plays a pivotal role in shaping students’ academic life. Autonomously motivated students use deep learning strategies [1], develop adaptive learning attitudes [2], engage actively in class [3], and have better academic performance [2, 4–6]. However, students face challenges maintaining their autonomous motivation within various curricula [7–9], which also holds true in the field of Health Professions Education (HPE) [10–14]. Some curriculum characteristics might hinder students from developing autonomous motivation. Curricula that are predominantly teacher-centered and fail to show the relevance between scientific concepts and practice could lead to a decline in motivation [10, 15]. Recognizing these hindrances is crucial to develop curricula that foster students’ autonomous motivation. Exploring ‘why’ such curricula stimulate students’ autonomous motivation and ‘how’ they achieve it may provide insights for the design and implementation of future curricula.

Prior to developing such curricula, it is essential to understand the concept of autonomous motivation. According to the Self-Determination Theory (SDT), autonomous motivation occurs when people engage in activities with a full sense of willingness, volition and choice [16]. Autonomously motivated individuals are more likely to engage in and persist with their behaviors because the behavior is self-determined and consistent with their intrinsic goals [17]. Autonomously motivated students report increased engagement, learning and well-being [18]. SDT posits three states of motivation underpinning autonomous motivation: intrinsic motivation (engage in activities because of interests, enjoyment and inherent satisfaction), integrated regulation (because of integration with the sense of self), and identified regulation (because of the personal importance and value of the activity). SDT distinguishes autonomous motivation from controlled motivation, which arises from the external reasons for participating in activities. Controlled motivation comprises two states: introjected regulation (engage in activities to avoid guilt or gain self-esteem) and external regulation (to avoid punishments or acquire rewards) [19]. In addition to autonomous and controlled motivations, SDT identifies amotivation as the least self-determined with the absence of any intention to perform [20]. Autonomous motivation is the desirable type of motivation as it has been consistently demonstrated to have associations with positive academic outcomes [18] and well-being [21].

Three pillars facilitate students’ autonomous motivation, namely autonomy, relatedness, and competence. They constitute the three basic psychological needs, the critical resources underlying individuals’ natural tendency towards self-organization and flourishing [22, 23]. Support of these three needs can help students to feel more autonomously motivated for learning [24]. Autonomy refers to a sense of initiative and ownership in one’s actions and is supported when a curriculum can provide choice and allow students to work at their own pace [25]. Relatedness concerns a sense of belonging and connection with others, such as peers and teachers. It is supported in environments that emphasize a warm, positive, and caring atmosphere [26]. Competence describes the need to experience a sense of mastery [18], efficacy in one’s actions [17], and the feeling that one can succeed and grow [18]. Students’ competence is satisfied within well-structured environments that provide optimal challenges, opportunities for growth [18], and explicit performance feedback [27]. The satisfaction of these needs stimulates students’ autonomous motivation because their behaviors are self-determined (autonomy), influenced by the others in an environment (relatedness), and aligned with effort (competence) [28]. However, when curricula fail to support these needs—e.g. when students feel controlled by grading systems, have no affinity towards peers and teachers, or are overwhelmed by cognitive load—they can hardly develop autonomous motivation [24].

There is a growing body of research studying the basic psychological needs and students’ autonomous motivation in their curricula. Ample studies revealed the positive associations between basic psychological needs satisfaction and learning environment elements [29], and between motivation and these elements such as feedback [30], learning approach [4, 14, 31, 32], and curricular subjects [33]. A few studies explored students’ experiences in their curricula and reported elements that enhanced students’ motivation, such as authentic situations and informal contact with peers [34], freedom of choice and teacher availability during discussion [35], relevance of course content for their study programmes [35, 36] and rationale provided in an autonomy-supportive way [37].

Although these studies offered insights into what curricular elements were related to students’ basic psychological needs and motivation, there is a lack of qualitative studies that reveal the underlying SDT mechanisms [38] and how and why elements associated with the SDT mechanisms may support or hinder students’ motivation. Some studies employed a combination of quantitative and qualitative methods but may not have provided a comprehensive understanding of the principles due to using observation tools not designed for SDT [39] or having collected answers from open-ended survey questions [35, 36, 40–42]. There is a need for qualitative studies with detailed educational innovations that support the basic psychological needs [18] and more clarification studies in HPE that address the question “Why or how did it work?” [43, 44]. Without understanding the underlying principles that stimulated motivation, it remains a challenge to integrate SDT constructs into a curriculum. Existing studies that did integrate SDT constructs in curricular design featured a single learning activity [25, 45] or focused on only one of the basic psychological needs [25, 46]. To sum up, there is a gap in understanding and implementing curricular principles that stimulate students’ basic psychological needs and autonomous motivation.

Given that student motivation had been largely ignored in the designing phase in HPE curricula [38, 47], we underscore the need to develop a curriculum that integrates the SDT constructs to support students’ autonomous motivation. Exploring the principles underlying what motivates students in such a curriculum may help teachers and curriculum designers to redesign their educational practices.

## The present study

We designed a so-called ‘Societal Impact Project (SIP)’ with three educational design principles: authentic learning, collaborative learning, and scaffolding. We expected these principles to support students’ basic psychological needs and stimulate their autonomous motivation.

The first principle was to foster authentic learning. Authentic learning is an instructional method that facilitates students’ learning by engaging them in tasks to solve problems [48, 49]. It has the potential to motivate learner participation [50], encourage students to actively construct knowledge [51], and enable autonomous learning behaviors by empowering students to take ownership of the problems [52]. Authentic activities possess characteristics such as real-world applicability, integration across the curriculum, and options for students to select appropriate levels of complexity and involvement [50, 53].

The second principle was collaborative learning. It describes a situation where interaction among people occurs and triggers learning [54]. It takes place when students have a common goal, share responsibilities, are mutually dependent, and reach agreement through open and collaborative interaction [55, 56]. It is positively associated with the feeling of relatedness because learners exchange information, work interdependently, and leverage each other’s contributions [57]. It contributes to the improvement of students’ academic motivation [58] as students take an active role in their own learning [58, 59], and group members can also help encourage individual motivation [58, 60].

The third principle focused on scaffolding. It refers to the temporary support for learners to complete a task that they otherwise may not be able to complete on their own [61]. The term scaffolding is becoming increasingly synonymous with support [62]. Scaffolding is highly effective in assisting learners to complete challenging tasks [63] and promoting learners’ motivation [62]. It can take different forms, for instance a teacher who provides prompts and feedback, who models the use of cognitive strategies by thinking aloud, and who offers support by presenting checklists and asking leading questions. Teachers in a learning environment that supports scaffolding should not direct learners but rather guide them while working on complex tasks and, therefore, deciding on the right type, amount, and time of support is crucial [64].

## Research question

This study aimed to answer the question “How does a SIP with characteristics of authentic learning, collaborative learning, and scaffolding support students’ autonomy, relatedness, competence, and autonomous motivation from students’ and teachers’ perspectives?”

## Methods

### Participants

We ran the SIP as an extracurricular project from November 2022 to June 2023, in total during a period of 32 weeks, at Maastricht University, the Netherlands. Students participated voluntarily and did not receive credits. First-year students in the bachelors’ studies of Biomedical Sciences and Health Sciences participated. All students from these two programmes were invited to an information lecture about the SIP, during which one of the authors (HS) presented the project. Students signed up by scanning a QR code at the end of the lecture or via the registration link shared by their course coordinators. Teachers from various university departments were recruited as ‘coaches’ for the SIP. These teachers had received university training in the Problem-Based Learning (PBL) approach and had experience as PBL tutors, which offered them the skills needed to provide support and guidance within the SIP. Hereafter, these teachers will be referred to as ‘coaches’. One of the authors (HS) invited them via email, introducing the SIP and explaining the coaches’ roles: guiding and facilitating student collaboration through supporting their basic psychological needs, as well as creating an autonomy-supportive learning environment. After confirming their participation, coaches received supporting materials, including a project overview, an explanation of the Creative Problem Solving (CPS) approach, and a project manual which outlined all activities during the SIP. They also attended a kick-off event where practical matters were discussed. Additionally, three coach meetings were organized during the project to address questions, discuss challenges, and share experiences. Coaches were invited to participate in all SIP activities and events, allowing for informal interactions.

### The SIP

**Fig. 1:**
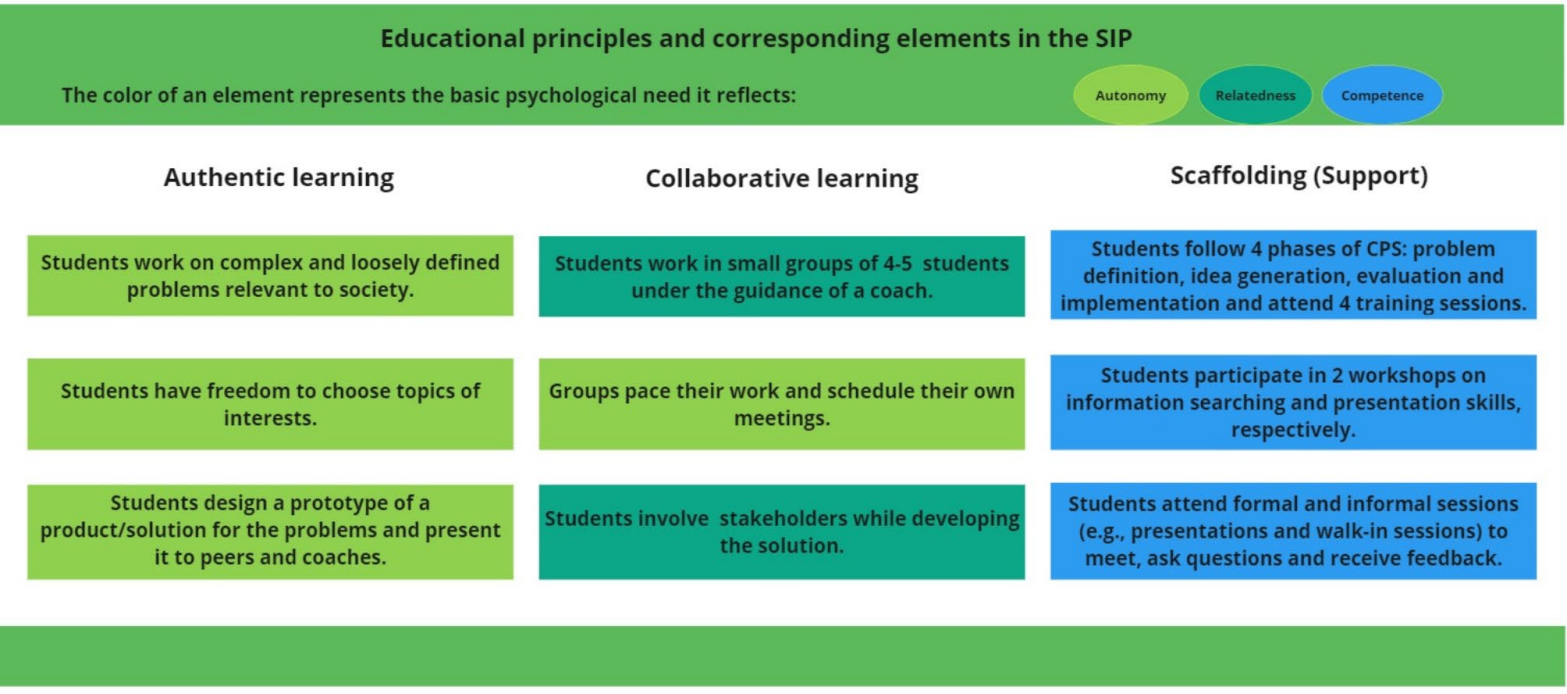
Educational principles and their corresponding elements in connection with the three basic psychological needs. *Note: One element can reflect more than one or two need(s) (e.g.*,* working as a group to involve stakeholders can facilitate relatedness and competence). The color of the elements reflects the most relevant need*

The aim of the SIP is to foster students’ autonomous motivation towards their studies through developing knowledge and skills in their fields and understanding the societal relevance of their studies. Three educational principles (authentic learning, collaborative learning, and scaffolding) interdependently support students’ basic psychological needs and motivation. We defined concrete practices that entail both the principles and the basic psychological needs (Fig. 1).

In SIP, students engaged in authentic learning activities and problems. For example, from the topics provided by coaches, they chose a loosely defined problem outlined in broad terms and was relevant to their study programmes and society. The problems were outlined in broad terms only to avoid detailing the scope, complexity or possible solutions. Students had the freedom to narrow down and decide the problem statements and the potential solutions of their interests. During the SIP, each group took the responsibility to plan their meetings, pace their progress, and attend workshops useful for their projects. Students developed collaborative learning through working in small groups, presenting and receiving feedback from peers and coaches, and involving stakeholders in developing their solutions. For scaffolding, students used an online system for accessing practical information, receiving announcements, and uploading materials. They followed the four phases of the CPS approach, which aims to foster students’ creativity and support them in developing solutions using divergent and convergent thinking [65]. Students received one training session on each of the four phases of CPS, namely problem definition, idea generation, idea evaluation, and implementation [66, 67]. In the first phase, students explored the literature, approached stakeholders, and defined the problems to be solved. In the second phase, they came up with as many ideas as possible regarding how to address the problem. Next, students evaluated and selected the most promising ideas. In the fourth phase, they reached an agreement on the chosen idea and implemented it [65, 67]. Complementing these training sessions, two workshops were offered during the first and last months of the SIP, focusing on information searching and presentation skills, respectively. Additionally, three walk-in sessions provided an informal space for students and coaches to meet, ask questions, and seek feedback. All groups presented their work twice in a format of their choices and received verbal feedback halfway and at the end of the SIP. Coaches had autonomy in guiding and supporting their groups to develop the solutions for the problems.

### Data collection

We conducted five focus group interviews with participating students and one focus group interview with coaches the week after the SIP finished. Students from the same groups were allocated to different focus groups, except in the case of scheduling conflicts. We developed semi-structured interview guides for both students and coaches (Appendix A and B) with structured, yet broad and open questions on students’ experiences of authentic learning, collaborative learning, scaffolding, and motivation during the SIP. To gain a comprehensive understanding of coaches’ experiences, the interview guide focused on three aspects: coaches’ motivation, perceptions of the integration of the educational principles in the SIP, and their experiences when working with the students. The latter two were pertinent to our research question. We asked the participants to draw a graph of their motivation throughout the SIP (Appendix C).

### Analysis

The focus groups were recorded and transcribed verbatim by a university-accredited company. We adopted thematic analysis and followed its six steps [68] (Table 1), discussed, and documented the coding strategies (Appendix D). Coding was both inductive (codes close to the data) and deductive (we used authentic learning, collaborative learning, and scaffolding as sensitizing concepts to analyze the data). Themes were organized latently to reflect connections and underlying meanings. We used Reproducible Open Coding Kit (Rock) [69] on R Studio for analysis because Rock is open-access and enables researchers to code, merge, and compare codes.

**Table 1:** Thematic analysis steps and author involvement

Step 1	Familiarizing with the data	• Proofread and check transcripts (YZ) • Discuss coding strategies and analysis procedures (DD, LA, YZ)
Step 2	Generating codes	• Independently code student focus group 1 and generate initial codes (LA, YZ) • Discuss and corroborate codes from focus group 1 and develop a coding framework (LA, YZ) • Continue to independently code student focus group 2 and 3 using the coding framework, add new codes to the coding framework (LA, YZ) • Merge and compare codes from focus group 2 and 3 (YZ) • Independently code student focus groups 4, 5 and coach focus group (YZ)
Step 3	Searching for themes	• Cluster and interpet codes (LA, YZ) • Use thematic maps to formulate sub-themes and compose initial themes (YZ)
Step 4	Reviewing themes	• Review thematic maps and discuss the representation of themes on the data set (DD, HS, LA, SEK, RAK, YZ) • Revise the thematic map and themes based on the feedback (YZ)
Step 5	Defining and naming themes	• Write narratives for each theme and select quotes (YZ)
Step 6	Producing the report	• Write the analysis and description of results (YZ) • Review writing and give feedback (DD, HS, LA, SEK, RAK) • Finalize writing (YZ)

### Reflexivity

The authors have different academic and professional backgrounds that might have influenced their perspectives. YZ is a PhD candidate researching undergraduate students’ motivation and is familiar with the relevant literature. She has experience in interviewing and teaching. YZ plans and organizes activities for the SIP but does not have any instructing or coaching role in the project. HS does research in movement sciences and educational sciences. His research on education focuses on improving student motivation, well-being, and curriculum innovation. LA is a researcher and university teacher and has been a tutor and mentor for more than 10 years. Her research focuses on reducing socioeconomic health inequalities. SEK is a researcher in the domain of anatomy and education and has experience as educational program leader, tutor, and mentor for over 20 years. HS and SEK were two of the coaches in the SIP but were not involved in any data collection from students. RAK is a medical doctor and researcher on inclusion and motivation in HPE. She is an acknowledged expert on SDT. DD is an educational scientist with expertise in instructional design. All researchers have experience with both quantitative and qualitative studies. The outsider positions (e.g. non-shared identity with participants as researchers and teachers) and insider positions (e.g. a shared identity with students as an academic with an interest in societal impact) may have impacted the analyses and interpretation of the data.

## Results

Forty-two students and nine coaches participated in the SIP, forming nine groups, each consisting of four to five students and one coach. These groups developed solutions for a variety of problems, such as developing an affordable diet low in highly processed foods, or understanding the relationship between poverty and lifestyles (Appendix E). Eighteen students and five coaches were interviewed in the focus groups. The five student focus groups consisted of three, four, two, five, and four participants, respectively. Student focus groups lasted on average 48 min, and the coach focus group 51 min. We identified three themes (Table 2) from the analysis of student and coach focus groups.

**Table 2:** Themes and subthemes

	Themes	Subthemes
Theme 1	Having freedom was motivating, but students needed support.	Having freedom to choose problems and self-pace their work were motivating for students.
		Having limited instruction and guidance left students feeling lost and demotivated, but this can be remedied by support.
Theme 2	Working in small groups could be motivating or demotivating, but communicating the difficulties with each other made the groups strong and motivated.	Actively collaborating and developing connections with peers and coaches were motivating.
		Talking about the problems that led to a dip in motivation facilitated group progress and motivation.
Theme 3	Societal relevant problems stimulated autonomous motivation and learning, but the link between the problems and students’ curriculum was not always clear.	Working on problems of value and practical meaning fostered students’ motivation and learning.
		It was challenging for students to see the link between these problems and the ongoing curriculum.

### Theme 1: having freedom was motivating, but students needed support

*Having freedom to choose problems and self-pace their work were motivating for students*. Students appreciated the freedom to choose from a wide range of problems and solutions that aligned with their interests. They also experienced a sense of freedom from planning meetings based on their own group pace. These characteristics gave students a sense of ownership of their projects.

*I think developing your problem statement is really helpful for motivation*,* as it’s really something of your own and you don’t want to butcher it*,* so you want to go forward with it because it’s something you kind of came up with. (Student 1*,* focus group 1)*

*She (coach) didn’t really push any specific directions into the final product. She let us decide ourselves…any idea we had*,* she’s like*,* “Yes*,* let’s do that. That’s very cool.” That contributed to our motivation a lot*,* I think. (Student 4*,* focus group 5)*

*However*,* having limited instruction and guidance left students feeling lost and demotivated*,* but this can be remedied by support.* With the freedom they received, students could feel lost and unsure of the expectations, decisions to take, or how to proceed in the face of setbacks. Feeling trapped within freedom led to a decline in students’ motivation. For example, a few training sessions had low attendance, as students were unsure whether attendance was mandatory. The low attendance caused by this uncertainty affected the motivation of those who were present for the sessions. Students needed guidance from their coaches, particularly in the initial phases of problem identification and idea generation when they had not yet formulated a concrete idea.

*When I go there and there are only seven people*,* I was a bit less motivated to go the next time because it was helpful if it was a bigger group (Student 1*,* focus group 2).*

*We had times that we had a bit of trouble with*,* how do we need to do this? How can we come up with something that would lead us to what we want to do?…There was a bit too much freedom. I guess a bit of guidance would be nice…I like that we have space to do what we want to do but if we have trouble with anything*,* we’re still first years*,* we don’t know that much really. It would have been nice to have some guidance on how we can approach this. (Student 2*,* focus group 5)*

*It’s great you have a lot of freedom. It is very broad*,* which is good because all the groups have very different topics and areas*,* but it would have helped to have a bit more structure within the group. So you kind of knew what way you should go*,* because at some point*,* we really just didn’t know what we should do. (Student 2*,* focus group 2)*

The crucial factors that supported students to navigate the freedom included the structured activities from the SIP (e.g., training sessions, timelines, and information on the online platform) and, most importantly, adaptive guidance from their coaches. Some students indicated that their coaches stepped in when they faced challenges (e.g., in reaching out to stakeholders) or felt stuck (e.g., when selecting ideas), which was also acknowledged by the coaches.

*It (training session) did really give some structure to the project*,* so we had something to work towards to…The training sessions*,* workshops*,* and deadlines really helped. We had a kind of a goal in mind up that until that point we can work on this*,* and after that we can go on to the next step, I would say. (Student 1*,* focus group 1)*

*She (coach) was really helping us to go through it (feeling stuck) and supporting us and helping with some ideas or some people we could talk to to get more information and that kind of stuff. (Student 1*,* focus group 2)*

*What I do is sometimes I give them an idea or say*,* have you looked at that or I send them a podcast*,* or I send them an interesting link and say*,* have a look at this (Coach 4).*

Some coaches were actively engaged with their groups at the beginning and gradually withdrew as they noticed the group’s progress and capability to lead themselves. This statement aligns with the coaches’ who intended to give the lead and responsibility to students. Coaches observed that in groups where students took initiatives, students updated coaches of their progress, invited coaches to meetings, and convinced coaches using the insights from stakeholder interviews. Some coaches stated that students struggled to take the lead in their projects; therefore, the coaches had to regularly check in with students.

*She (the coach) let us have our freedom*,* but if we needed help*,* she was always there to help us*,* especially in the first few weeks. Then as we got more off the ground*,* she backed off a bit…In the first few weeks that definitely helped with keeping the motivation up… (Student 3*,* focus group 1)*.

*Maybe she noticed that we are just working on our own and we are functional. She just let us be and always was there for us if we needed something. (Student 1*,* focus group 5)*

*…the students took the initiative. They did not rely too much on me*,* but they came up with their own ideas*,* and they did a lot of work and just came back to me asking for my input about what they already did. I liked that a lot. I was not in the lead. They were in the lead and that was nice. (Coach 1)*

*I wanted to give them the initiative*,* but they didn’t really take it*,* so I tried to contact them frequently until the mid-presentation…It improved a little bit after because I tried to put some more effort into it again. I was like*,* okay*,* maybe I should reach out more often and maybe I should help them more and more. Unfortunately*,* it didn’t work…I kind of failed to give them the lead or to stimulate them to take the lead*,* I guess. (Coach 2)*

### Theme 2: working in small groups could be motivating or demotivating, but communicating the difficulties to each other made the groups strong and motivated

*Active collaboration and developing connections with peers and coaches were motivating.* Students were more willing to participate in the project if they were friends with peers and had informal contact with coaches. Their motivation decreased if the group members contributed unequally or did not meet frequently, which affected the group’s progress from the coaches’ perspectives.

*The people there are my friends*,* so I’m excited to see them*,* and I’m excited to work on the project. That’s what helped the motivation*,* I would say. (Student 2*,* focus group 5)*

*…we got to go together at our mentor’s house*,* and we were cooking together. This was really creative and enjoyable. I guess we were all quite motivated. (Student 4*,* focus group 5)*

*…or you notice that you’re doing a lot of work and then some of the other members aren’t*,* it can be very demotivating because you put in that much work and you’re not sure what you’re putting that work in for. (Student 3*,* focus group 1)*

*I think it was mostly the fact that we were not meeting up and really talking to each other that much during that time. We did not know what everyone was thinking and we were not communicating very well. I would not say an annoyance at the moment*,* but it was definitely not something very positive and motivating. (Student 1*,* focus group 4)*

*We didn’t connect that often anymore and then you lose a little bit of track. In the end… we had more contact again and then we improved (Coach 1).*

*Talking about the problems that led to a dip in motivation facilitated group progress and motivation.* Both students and coaches shared that being able to discuss the difficulties the groups encountered made the groups stronger, such as unequal contributions and a lack of interaction. Having effective collaboration and friendly connections made it easier for students to communicate the difficulties as they felt comfortable to openly share their views. Being able to honestly discuss challenges with each other helped in tackling the challenges and gaining back the motivation, while a lack of such discussion could lead to students leaving the project for good.

*I think it was good that it happened*,* the motivation drop*,* I think that made us stronger as a group. You know each other and then if [name] has a drop and I say*,* “Come on*,* we need to do this and this.” Then did it. (Student 1); It really helps to pull each other up. That also helps with your own motivation*,* because you feel like if you see someone else less motivated*,* you feel like I need to pull them up*,* so I need to be more motivated as well. (Student 2*,* focus group 2)*

*…There were some group troubles*,* and some people thought they were doing more than the others. They talked about that with each other. It remained a really serious group that said*,* well*,* this is a problem*,* so we are going to discuss it. A lot of people were sick all the time*,* and they talked about it. They didn’t share that with me but amongst each other. They had more understanding of each other’s absence and then the group process improved again. (Coach 5)*

*We really noticed when the motivation went down when people started communicating less. There was less interaction. When something was said in group chat*,* for example*,* people were less responsive because they didn’t feel very motivated. We had quite a meeting about that. We really discussed it*,* really communicated about it. After that*,* it went way better. (Student 2*,* focus group 2)*

### Theme 3: societal relevant problems stimulated motivation and learning, but the link between these problems and the students’ curriculum was not always clear

*Working on problems of value and practical meaning fostered students’ motivation and learning.* Students recognized the application of knowledge, foresee the impact of their work, and picture a future career. These aspects contributed to students’ motivation.

*…when you’re just studying*,* it’s all theoretical. You’re kind of thinking “I’m studying this*,* but for what?” I don’t see anything happening yet. The moment you’re busy with the project*,* you see*,* you can implement this*,* and it can have a positive effect, and it can bring something to society. (Student 2*,* focus group 2)*

…*because our project involves a lot of talking to specialists and experts*,* it is very helpful to see what our potential future is if we decide to go that way. We know what the representation of it is. That motivates us all. (Student 3*,* focus group 4)*

*At the beginning of the study of health sciences*,* I was blank and just from high school*,* and I think it helped me to achieve for myself a broader view of what I can do and what I can mean for people…It has a good impact on my motivation. (Student 2*,* focus group 3)*

Students and coaches agreed that students gained knowledge related to their topics and harnessed a broad spectrum of generic skills. These skills included planning, networking, communication, teamwork, presenting, time management, professional behavior, problem solving, etc.,— all of which were acknowledged as useful for students’ own studies or their future careers. Students felt proud to present and disseminate their work during the presentations or at the university.

*With my background knowledge of biomedical sciences*,* I can actually have a conversation with the scientists…I wanted to just develop myself within this project and develop my knowledge of medicine. It’s great for making connections with scientists for my future. (Student 3*,* focus group 4)*

*…I do think it taught me some valuable skills in the field of societal impact*,* contacting other people*,* networking in general*,* and also setting up a project. In that sense*,* it really helped me to get an insight into how those things might go. I see it as a nice addition. (Student 3*,* focus group 1)*

*What they learned in the SIP project is to distribute work*,* make appointments*,* keep deadlines*,* take initiative*,* and contact people. (Coach 4)*

*I think it maybe just taught me to be a lot more confident because you have to go out and do things and push yourself out of your comfort zone*,* in order to get things done. This helped my confidence in biomeds (student’s study programme)*,* for example*,* in just being in a tutorial and being a chair*,* or just if you’re in a group project and no one’s really doing their work. It helped me to actually take the initiative. (Student 3*,* focus group 5)*

*We were proud that we had some product to show during the end presentation*,* and it’s still something we’re working on*,* and we’re planning on doing a little stand somewhere in university*,* so the others can see your product as well. I think this was very fun to do. I’d say in the end*,* in these weeks*,* motivation is quite high…to just finalize the project and show our peers. (Student 2*,* focus group 1)*

Although students found their projects helpful for acquiring new knowledge and skills related to their studies, *it was challenging for them to see the link between their SIP problems and the ongoing curriculum.* They understood that the wide array of topics could add to their knowledge in their fields of study at large, yet they also wondered about the relevance of these topics and approaches to their ongoing curricula. One of the coaches shared the same view.

*It’s usually pretty related depending on the topic you chose*,* but it’s always nice to have extra information*,* to have extra knowledge about certain topics*,* even if it’s about the environment*,* even if it’s about soya milk*,* for example*,* things like that…Everything is all related to biology eventually. All the extra knowledge you can get is always useful. (Student 4*,* focus group 4)*

*I feel like a lot of people would enjoy if this was made somehow more scientific with some lab practice*,* or maybe some topics that can be also more practically implemented…this would be very interesting for everybody and maybe also increase the motivation of people because it would more correlate with their real-life studies. (Student 4*,* focus group 5)*

*There was*,* of course*,* a huge societal arm*,* but the research knowledge arm is less pronounced*,* and I would like to link it more to the programme that they are studying. (Coach 1)*

## Discussion

This study offered insights of how and why some elements of SIP, designed following the three educational principles, facilitated or hindered students’ motivation. The findings also provided suggestions of how to integrate and apply these principles in the learning environments.

To foster autonomous motivation, scaffolding should be in place while providing autonomy to students. Students experienced autonomy-supportive elements in the SIP such as defining problems of interests, regulating group learning progresses, working at their own pace [41], and taking ownership and initiative in their activities. This conclusion fits with the SDT, emphasizing that curricula should foster students’ feelings of autonomy, relatedness, and competence [18]. However, autonomy without sufficient and adaptable support left students lost and stuck, which led to a decline in motivation. This deomstrated the challenge of faded scaffolding, a transfer of responsibility from the scaffolder (i.e., teacher and the learning environment) to the learner as the learner is becoming capable of independently performing an activity [62]. Scaffolding should be adapted to the students’ level of self-regulation and not purely rely on students calling for help since not all students can regulate their learning and seek support when necessary [62, 70]. It is important to confront students with problems of increasing complexity and to offer a high level of support and guidance at the start which is then gradually withdrawn when students become more competent in solving problems of a specific level of complexity [71]. This could be particularly important for students in the SIP, as they were first-year university students who may still find the transition from school to university overwhelming [9]. Pevious studies exploring students’ motivational experiences in their learning environments also underscored the need of support along with autonomy [34, 72, 73]. Providing guidance along with autonomy aligns with the SDT term autonomy with structure, which can be achieved through defining explicit expectations, maintaining consistency in rules and guidelines, and offering support for engagement and feedback [18]. Good support scaffolds learning, and the combination of high autonomy with support is associated with high autonomous motivation [74]. Autonomy and structure do not oppose each other. Instead, “they can, and should, exist side-by-side in a mutually supportive way” [74, 75].

Collaborative learning cultivated a sense of relatedness when students worked closely and effectively in small groups. Building rapport with peers and teachers facilitated autonomous motivation as students were inclined to internalize and embrace the values of those with whom they felt connected and a sense of belonging [3]. When students felt connected with their peers, they felt comfortable discussing their difficulties openly, which helped them regain their motivation. This finding was consistent with a previous study that peer connectedness had a positive association with academic resilience and student hope in the face of challenges [76]. In addition to feeling connected with peers and coaches, effective collaboration was pivotal to student’s autonomous motivation, such as active participation [26], teamwork contributions [35, 42], and team communication [34]. Students also reported challenges when working with each other, such as disagreements, unequal contribution, and a lack of communication and interaction. These issues have been previously identified as common hurdles in implementing collaborative learning [77]. Aligned with findings from other studies, these experiences decreased students’ motivation [34, 41]. Teachers should be able to address problems of group dynamics and evaluate the group functioning on a regular basis to facilitate collaborative learning [56].

Authentic problems enhanced student’s autonomous motivation and learning. Students acknowledged the rationale and value of the authentic problems [34, 45, 78, 79], that is, making contributions to real-world practice [34, 40]. When students recognized the value of their subjects, they autonomously chose to study them, thus shifting their motivation toward self-determined motivation [26]. Authentic problems also fostered students’ learning of knowledge and skills. As students presented and defended their solutions, they articulated their understanding publicly, improving knowledge construction and laying a foundation for future learning [80, 81]. By involving stakeholders, students acquired knowledge from a wide array of sources, which enabled them to understand how knowledge supports problem solving [81]. Although authentic problems were perceived as motivating and supportive of learning, students found it difficult to see the link between these problems and their ongoing curriculum. Providing a clear explanation by coaches of how these problems may contribute to students’ studies (i.e., knowledge and skills are transferable to their own curricula) can help bridge this gap. Additionally, developing open, flexible, authentic curricula that respond to the rapid changes and unpredictable challenges may also aid students to understand the link between a curriculum and the society [82].

### Strengths and limitations

The study has two major strengths. Firstly, we applied three theoretical principles from literature that support autonomous motivation to design and develop this project. By explicitly describing the design, analysis, and evaluation of the study, we provided insights for curriculum designers to develop similar or adapted motivation-stimulating courses. Secondly, we incorporated the perspectives of both students and coaches, which helped us to gain a rich understanding of students’ motivation-related experiences.

Our study has some limitations. Firstly, the study took place in a specific context; all students and coaches were from the same university and had experience with small-group teaching, which might limit the transferability of the findings to other contexts. Secondly, this study focused on students’ and teachers’ self-perceptions, and we did not include perspectives of other stakeholders, such as training instructors and external stakeholders whom students contacted.

### Future directions

We propose two directions for future research. Firstly, we recommend replicating educational projects in other settings, institutions, and study programmes on diverse subjects. Secondly, researchers should consider including perspectives from other stakeholders, for instance external stakeholders and curriculum coordinators. This may help to gain a comprehensive picture of students’ motivation and improve the design of the project.

### Practical recommendations

Based on our finding that SIP stimulated students’ autonomous motivation, we recommend that teachers and curriculum designers incorporate elements supportive of basic psychological needs into the learning environments. We highlight the importance of providing scaffolding and structure to help students navigate autonomy. Scaffolding requires teachers to identify students’ needs and provide support that corresponds with students’ ongoing progress. Autonomy-supportive environments do not equal independence, which is working without help [83]. We recommend that teachers offer timely and constructive feedback and step in when noticing students’ getting stuck to help students find a balance between freedom and guidance. Curriculum designers can consider evaluating and improving the structured activities, so they provide clear timeline and guidelines, describe expectations for both students and teachers, and offer platforms with relevant information and resources.

We recommend that teachers and curriculum designers explore ways to facilitate small groups to not only work closely, but also effectively. Merely working in small groups with peers and coaches does not guarantee a feeling of connection, whereas effective collaboration stimulates a sense of belonging to a group. Having frequent meetings, active discussions, and regular check-ins facilitates collaboration and enhances motivation. Teachers can encourage open conversations and show openness to genuine discussions [84]. Curriculum designers may consider organizing informal events and the introduction of guidelines or workshops on team collaboration and communication.

Teachers can help students identify the practical value, career prospects, and useful skills when tackling authentic problems. There is a need for curriculum designers to first investigate students’ needs and interests when attending a curriculum and then define the level of relevance of authentic problems to students’ ongoing curriculum. Teachers should be available to discuss the relevance of authentic problems with students.

## Conclusion

In this study, we developed and evaluated how and why SIP, designed using the principles of authentic learning, collaborative learning, and scaffolding, supported students’ basic psychological needs of autonomy, relatedness, and competence, as well as their autonomous motivation. Some elements of the SIP contributed to students’ autonomous motivation. The results underscore the importance of providing autonomy with scaffolding, facilitating relatedness with open communication, and fostering competence with authentic and societal relevant problems. Striking a balance between offering autonomy and support is crucial, so as explaining the relevance between real-life problems and their ongoing curricula to students. This study provided insights for designing curricula that foster students’ autonomous motivation.

## Electronic Supplementary Material

Below is the link to the electronic supplementary material.

Supplementary Material 1

## Data Availability

The datasets used and/or analysed during the current study are available from the corresponding author on reasonable request.

## Abbreviations

HPE: Health professions education
SDT: Self-determination theory
SIP: Societal impact project
PBL: Problem-based learning
CPS: Creative problem solving
