# Supplementary material for: Fostering university students’ autonomous motivation through a societal impact project: a qualitative study of students’ and teachers’ perspectives
Source: BMC Med Educ. 2024 Dec 20;24:1503. doi: 10.1186/s12909-024-06494-9 (PMC11660546; doi:10.1186/s12909-024-06494-9)
Supplement: Supplementary file 1 — Supplementary Material 1 [file 12909_2024_6494_MOESM1_ESM.docx]

Appendix A: Interview guide questions for students

1. Can you tell us a bit about yourself (name, age, and study programme)?
2. Can you use this graph (Appendix C) to indicate how your motivation changed during the SIP? Could you explain what happened for the highs and lows and how this caused a change in motivation for SIP?
3. You worked on a societal problem that you chose and defined yourself, what do you think of it? How and why did it enhance or hinder your motivation for SIP? Can you give some examples?
4. What do you think of the support you received during the SIP? Could you give some examples? How did this support or hinder your motivation for SIP and why ?
5. You worked in small groups with a coach, how were your experiences? How and why did this experience support or hinder your motivation for SIP? You also met other groups and stakeholders, what did you think of these experiences? How and why did they support or hinder your motivation?
6. How do you think your experiences in the SIP impacted (or not) your motivation for your own study programmes? Can you give some examples and explain why?
7. How and why was SIP a useful learning experience for you (or not)? Can you give some examples of what was helpful (or not) for your learning and why?
8. Do you have anything to add about your experiences in SIP? What about the SIP did you like or dislike and why? What can be improved to enhance your learning/motivation?

Appendix B: Interview guide questions for coaches

1. Can you tell us a bit about yourself (name, age, and teaching experience)?
2. Can you use this graph to indicate how your motivation for SIP changed during the project? Could you explain what happened for the highs and lows and how this caused a change in motivation and why?
3. You worked on societal problems that you chose and defined later together with students, what do you think of it? How and why did this experience enhance or hinder your motivation for SIP? Can you give some examples and explain why?
4. What do you think of the support you received as a coach during the SIP? Could you give some examples? How did the support foster/hinder your motivation for SIP?
5. You worked in small groups with your students, how were your experiences and how did these experiences support/hinder your motivation for SIP? As a coach in SIP, you also had opportunities to meet other coaches, what did you think of this experience and how did it support/hinder your motivation for SIP?
6. Do you have anything to add about your experiences in SIP? What do you like about the SIP? What can be improved to facilitate your motivation?

Appendix C: Graph example as focus group conversation starter


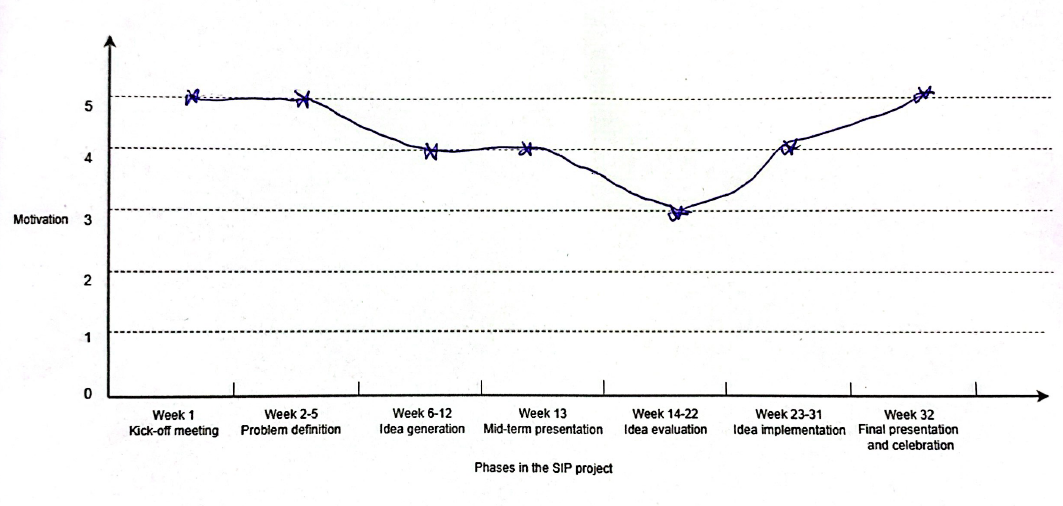


Appendix D: Coding strategies

YZ and LA discussed and used the following coding strategies across four meetings:

1. Code as close to the text as possible so it is easy to identify, discuss, and have an overview of the data. However, we acknowledge that participants’ responses could be influenced by the semi-structure interview questions. Therefore, it might reflect a deductive approach.
2. Code based on meaningful units.
3. Have one code per line.
4. Add the direction of the code, in other words, the relation between motivation and participants’ examples/behaviors/words based on the context participants provided. For example:

look at other groups🡪motivation down looking at other groups

concreate idea🡪motivation up with concrete ideas

*I'm just at the very bottom of the scientific world, and what does this mean for society?*

*Codes: [[motivation up because of curiosity of bachelor’s program’s relevance to society]]*

1. Code the quotes even if they are not directly related to the research question.
2. Do not make new codes if the same participant made a same statement.
3. Do not repeat previous codes when coding the first focus group, always use new codes close to the data even it has similar notations with previous code, e.g., ‘come to work’ and ‘ideas rolling’ indicate similar message, but we still use two separate codes.
4. Create a coding framework based on discussed codes from focus group 1. Use the codes from the framework to code other focus groups. Add new codes to the framework and mark from which focus group the codes were generated.
5. When using the coding framework, add information (such as examples) to the same code if needed. Keep using the same codes if it’s mentioned by different students.
6. We used both assimilations (integrating data that align with the existing interview questions and theories) and accommodation (accepting data that do not fall within the existing interview questions and theories) while coding.

Appendix E: Topics of SIP groups

| **Group** | **Project** |
| --- | --- |
| **1** | Breaking sedentary habits of students by raising awareness among architecture students |
| **2** | How to change the lifestyle of families in Maastricht that live in poverty to improve their health? |
| **3** | Integrating a course about climate change in the Bachelor’s programme of Biomedical Sciences at Maastricht University |
| **4** | Podcast: Sharing the breakthroughs in the field of regenerative medicine |
| **5** | ‘Project Hummus’: Implementing plant-based milk at Maastricht University café to promote healthy and sustainable diet |
| **6** | Developing a recipe book that help avoids highly processed food and the associated health risks |
| **7** | ‘Sitting is new smoking’: Change students’ sedentary lifestyles |
| **8** | ‘Risk it’: Understanding health risks |
| **9** | Antibiotic resistance: Close to home or at a distance? |
